# Problem-Based Learning in Pharmaceutical Education: A Systematic Review and Meta-Analysis

**DOI:** 10.1155/2014/578382

**Published:** 2014-02-19

**Authors:** Tais F. Galvao, Marcus T. Silva, Celiane S. Neiva, Laura M. Ribeiro, Mauricio G. Pereira

**Affiliations:** ^1^Faculty of Medicine, University of Brasilia, P.O. Box 4472, 70904-970 Brasilia, DF, Brazil; ^2^Getulio Vargas University Hospital, Federal University of Amazonas, 69020-170 Manaus, AM, Brazil; ^3^Faculty of Medicine, Federal University of Amazonas, 69020-160 Manaus, AM, Brazil; ^4^University Hospital of Brasilia, Pharmacy School, University of Brasilia, 70830-200 Brasilia, DF, Brazil; ^5^Department of Logistics, Executive Secretary, Ministry of Health of Brazil, 70058-900 Brasilia, DF, Brazil

## Abstract

*Objective*. To assess the effects of problem-based learning (PBL) on the learning achievements of pharmacy students. *Methods*. We searched for controlled studies that compared PBL to traditional learning in pharmacy courses (graduate and undergraduate) in the major literature databases up to January 2014. Two independent researchers selected the studies, extracted the data, and assessed the quality of the studies. Meta-analyses of the outcomes were performed using a random effects model. *Results*. From 1,988 retrieved records, five were included in present review. The studies assessed students' impressions about the PBL method and compared student grades on the midterm and final examinations. PBL students performed better on midterm examinations (odds ratio [OR] = 1.46; confidence interval [IC] 95%: 1.16, 1.89) and final examinations (OR = 1.60; IC 95%: 1.06, 2.43) compared with students in the traditional learning groups. No difference was found between the groups in the subjective evaluations. *Conclusion*. pharmacy students' knowledge was improved by the PBL method. Pharmaceutical education courses should consider implementing PBL.

## 1. Introduction

Problem-based learning (PBL) is an educational method focused on self-directed learning, small groups discussion with facilitators and working through problems to acquire knowledge [1]. It was first implemented in medical education in the 1960s [2]. This method can be an important tool in healthcare career education, in which students learn by working with real-life cases. In a PBL course, the instructor helps the students develop problem solving skills, self-directed learning, collaboration skills and intrinsic motivation, which led students identify what they know, what they need to know, and how and where to access new information they need [1, 3, 4]. It is expected that professionals will feel more prepared following PBL when faced with similar situations in the workplace.

PBL has also been used in pharmaceutical education courses, and numerous published reports describe the resulting experiences with this educational method [5]. However, the benefits of PBL for graduate and undergraduate pharmacy students have not been clearly shown. Thus, the aim of our study was to assess the effects of PBL in pharmacy education through a systematic review of the literature with meta-analysis.

## 2. Methods

### 2.1. Protocol

The current review was registered on the International Prospective Register of Systematic Reviews (PROSPERO), registration number: CRD42012002088.

### 2.2. Eligibility Criteria

We considered eligible controlled studies that compared the use of problem-based learning (PBL) methods in graduate (continuous education or postgraduation) or undergraduate (college) pharmacy education courses to the use of traditional methods. The outcomes of interest were the effects on students' learning evaluations.

### 2.3. Information Sources

We searched the MEDLINE, Embase, Scopus, Cumulative Index to Nursing and Allied Health Literature (CINAHL), ISI Web of Science, Education Resources Information Center (ERIC), Academic Search Premier, Wilson Education Full Text, ProQuest, Literature in the Health Sciences in Latin America and the Caribbean (LILACS), and Scientific Electronic Library Online (SciELO) databases. To identify potentially eligible studies, we also hand-searched the Pharmacy Education Journal, the website of the International Pharmaceutical Federation, and references from relevant articles. The last search was performed in January 2014.

### 2.4. Search Strategy

We used the following strategy for MEDLINE (via PubMed): (“problem-based learning”[mesh] or “problem-based learning”[tiab] or “problem based learning”[tiab] or “problem-based curriculum”[tiab] or “problem-based curricula”[tiab] or “experiential learning”[tiab] or “active learning”[tiab] or “problem solving”[mesh] or “problem solving”[tiab]) and (“pharmacy”[tiab] or “pharmacist”[tiab] or “pharmaceutical”[tiab] or “students, pharmacy”[mesh] or “schools, pharmacy”[mesh] or “education, pharmacy, graduate”[mesh] or “education, pharmacy”[mesh]). Variations of this strategy were applied when searching other sources.

### 2.5. Study Selection and Data Collection Process

Two researchers (CN, LR) independently reviewed the retrieved studies. Disagreements were resolved by consensus or by a third reviewer (TFG). CN and LR extracted the data and TFG confirmed the extraction.

For crossover design studies, only the results prior to the crossover were included to eliminate the risk of measurement bias from the residual effects of the specific methods.

We designed a data extraction sheet to collect the relevant data from each study, including country, year, type of allocation, funding source, sample size, type of course, intervention duration, intervention description, and subjective and objective evaluations results. We contacted the authors of the studies as needed to obtain relevant data not included in the reports.

### 2.6. Quality Assessment

For this study, we only included studies that had comparison groups without systematic differences between the groups in the analysis. We considered random assignment to teaching methods an indicator of a high quality study. If the groups were formed by other means, we assessed baseline characteristics to determine if there was a selection bias that could favor any group of students. We also reviewed the losses in planned follow-up procedures to assess if there was any performance bias. Any study that did not meet the quality criteria was not included in this review.

We did not consider allocation concealment (to maintain confidential the information of which group each student belonged until the end of the study) and blinding (not knowing to which group each student belonged) in the quality assessment of the studies because these procedures are not feasible in educational research.

### 2.7. Data Analysis

The primary outcome was the standardized mean difference (SMD) of objective evaluations of learning. For better interpretation of the SMD, the odds ratio (OR) was further calculated using the equation ln(OR) = *π*/√3 × SMD [6, 7].

The meta-analyses of SMD were grouped by the random-effects Mantel-Haenszel model and are presented with 95% confidence intervals (95% CI). Heterogeneity of the results was estimated by the *I* ^²^, *τ* ^2^, and *χ* ^2^ (*P* > 0.10) tests, and the risk of publication bias was assessed by inspection of funnel plot asymmetry.

## 3. Results

**Figure 1:**
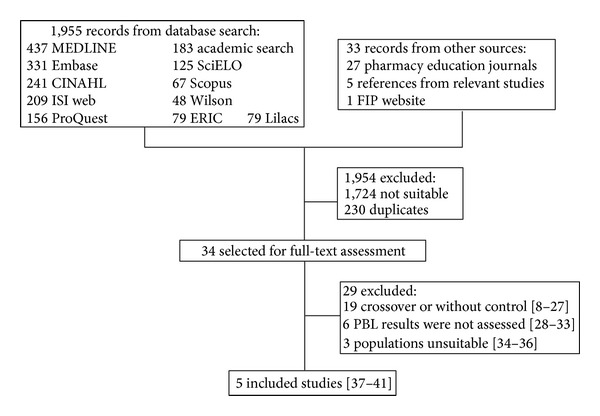
The search, selection, and inclusion process used in the study.

The literature search retrieved 1,988 articles, of which 34 articles were selected for full-text assessment [8–41]. Of that group, five studies were included in this review [37–41]. The flow diagram (Figure 1) illustrates the steps taken to select the studies for the current research.

### 3.1. Study Characteristics

Table 1 shows the characteristics of the included studies. Five studies measured the results of the PBL method through objective methods, although one study [40] used only a subjective evaluation involving a questionnaire designed to assess graduating students' perceptions about their preparation for practice. Another study used both objective and subjective methods through final examinations and surveys to assess the students confidence in performing certain practice activities, respectively [37].

The results of the studies were presented as means and standard deviations, using different rating scales. Two studies included a third group of students that was not of interest to this review: a group without intervention and a group in a transitional program using both traditional and PBL methods [39, 40]. Two studies reported losses from the initial sample; one study sent questionnaires to 186 students but finished with 137 students that completed the survey [40]. The other had five losses in the PBL group and four in the traditional curriculum group, and these were explained by refusal to participate, rural internship and scheduling conflicts [37].

Three studies formed groups by randomization [37, 38, 41]. One study assessed the students in the classes before and after PBL implementation and another one compared two courses held at different hospital pharmacies [39, 40]. There was no observed tendency in the selection of study methods and procedures in either group or during follow-up in any study.

### 3.2. Outcomes

**Figure 2:**
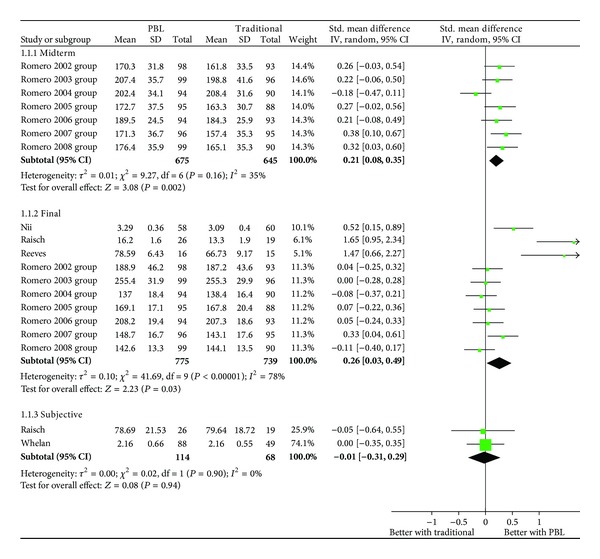
Results of the objective evaluations (final and midterm exams) and subjective evaluations.

The meta-analysis of both the objective assessments (midterm and final examinations) and subjective assessments is presented in Figure 2. While the final and midterm results significantly favored the PBL group, no difference was observed in the subjective evaluations. a Significant heterogeneity was found in the final exam results (*I* ^²^ = 78%). The sensitivity analysis revealed that two studies were the major contributors to this result [37, 39], and no quality limitation was found in the studies.

The OR estimates were also statistically significant for the midterm exams (OR = 1.46; IC 95%: 1.16–1.89) and for the final exams (OR = 1.60; IC 95%: 1.06–2.43), which indicates a better performance by PBL students compared with those participating in traditional learning methods. For the subjective assessment, no difference was found between the comparison groups (OR = 0.98; IC 95%: 0.57–1.66).

Despite the small number of studies examined, we assessed the funnel plot asymmetry and found that there was no suggestion of a risk of publication bias.

## 4. Discussion

The PBL pharmacy students performed better in academic examinations than the students in the traditional learning method group. Subjective evaluations of the students did not differ between the two groups. The findings indicate that the confidence in learning was similar between the two groups, while performance on course assessments was better in PBL. No study reported professional activity-related outcomes. To our knowledge, this is the first systematic review with meta-analysis of the effects of PBL in pharmaceutical education.

The present findings for pharmaceutical education are consistent with recent research in nursing [42], medical [43], and dental education [44]. This makes PBL the preferred education method for healthcare courses. The main barriers for implementing PBL are teacher and staff training and a necessary reduction in class size, which could increase the cost of pharmacy education [45, 46]. Research in the field shows, however, that PBL and traditional curriculum costs are comparable, and the main difference is the way teachers and other faculty personnel carry out their duties, because the PBL program requires greater engagement with students [47–49].

Because PBL encourages students to think about and solve real problems, it is likely to aid their use of knowledge in clinical situations, help develop their clinical reasoning, and encourage self-directed learning throughout their professional careers [4, 46]. In healthcare fields, these skills are highly desirable and valuable.

The present results are based on a small number of studies that may not reflect the diversity of pharmaceutical education in different contexts. To avoid missing relevant studies, we followed a registered protocol that comprised sensible searches in the major information databases and included studies conducted in both graduate and undergraduate pharmacy courses. To minimize the risk of bias, only studies of acceptable quality were considered. Another limitation of our review is the absence of studies that assessed effects on the professional achievements of pharmacists who experienced the PBL method compared with the achievements of those who were given traditional methods of instruction. The current results demonstrate that PBL students perform better academically; however, we cannot infer that this improvement results in better professionals.

Future research should prioritize experimental designs that assess outcomes that are more directly associated with professional effectiveness and more valuable in the workplace. More evidence in the field could be provided—without substantially increased costs—by recording and reporting the results of the implementation of PBL methods.

## 5. Conclusion

The PBL curriculum seems to improve the academic performance of pharmacy students when compared to the traditional method of instruction. Directors and teachers of pharmacy graduate and undergraduate courses should consider gradually introducing PBL methods into their programs. Reporting the results from such initiatives is likely to improve the quality of the existing evidence in support of PBL methods.

## Figures and Tables

**Table 1:** The main characteristics of the included studies.

Study	Country	Dates of research	Population	Random allocation	Type and length of course	PBL intervention (*N*)	Control intervention (*N*)	Outcomes
Raisch et al. 1995 [37]	USA	1993-1994	Undergraduate students	Yes	8 weekly sessions of two hours during externship	Small group discussion of cases led by facilitator and problem solving (26)	Externship with no modifications (19)	Objective and subjective evaluations

Nii 1996 [38]	USA	1994-1995	Graduate students (PharmD)	Yes	2 semesters in the third year preceding clerkship	Small group discussion of cases led by facilitator (58)	Didactic lectures (60)	Objective evaluation (grade point average)

Reeves and Francis 2002 [39]	United Kingdom	1997-1998	Pharmacists	No	6 months of continuing education	Adverse drug reactions cases in the context of a clinical scenario (16)	Didactic lectures (15)	Objective evaluation (multiple-choice questions)

Whelan et al. 2007 [40]	Canada	1998, 2001-2002	Undergraduate students	No	Entire graduation course	Small group discussion of cases led by facilitator and problem solving (127)	Didactic lectures (59)	Subjective evaluation (survey of perception of preparedness)

Romero et al. 2010 [41]	USA	2002–2008	Graduate students (PharmD)	Yes	4 weeks of PBL during the first semester of PharmD curriculum	Small group discussion of cases led by facilitator and problem solving (675)	Didactic lectures (645)	Objective evaluation (midterm and final tests)

Notes:

*N*: number of students.

PBL: problem-based learning.

PharmD: doctor of pharmacy (professional doctor degree).
